# Translation, cultural adaptation, and descriptive psychometric pilot evaluation of the Swedish Cancer Support Source-caregiver

**DOI:** 10.1186/s12913-026-15491-1

**Published:** 2026-08-31

**Authors:** Maria Samuelsson, Jenny Jakobsson, Benjamin Maus, Carl Magnus Olsson, Marie Louise Möllerberg

**Affiliations:** 1 Faculty of Health and Society, Department of Care Science, Malmö University, Jan Waldenströms gata 25, Malmö, Sweden; 2 Faculty of Technology and Society, Department of Computer Science and Media Technology, Malmö University, Malmö, Sweden; 3 CaRe – Cancer and Illness Rehabilitation Research Group, Malmö University, Malmö, Sweden; 4 Sustainable Digitalization Research Centre, Malmö University, Malmö, Sweden

**Keywords:** Anxiety, Caregivers, Depression, Needs assessment, Neoplasms, Psychometrics, Reproducibility of results, Stress, Psychological, Surveys, Questionnaires, Translating, Validation study

## Abstract

**Objective:**

This study aimed to translate the CancerSupportSource-Caregiver (CSS-C), culturally adapt it, and conduct a descriptive pilot evaluation of the psychometric properties of the Swedish version in a sample of Swedish family members of persons diagnosed with cancer.

**Method:**

Translation and cultural adaptation involved forward-backward translation by professional translators in collaboration with the research team and assessments of comprehensibility and face and content validity by two expert panels comprising family members of persons diagnosed with cancer and healthcare professionals, respectively. The psychometric properties, validity, and reliability of the Swedish version were pilot evaluated using classical test theory among a sample of 30 adult Swedish family members of persons diagnosed with cancer.

**Results:**

Based on requests from the expert panels, and supported by empiricism, the CancerSupportSource-Caregiver was adapted to the Swedish context, and five items were added. Thus, the Swedish version comprises 24 items. The face and content validity of the Swedish CancerSupportSource-Caregiver were satisfactory, and the convergent validity was promising. Moreover, the psychometric pilot evaluations indicated high data quality, overall satisfactory targeting, and internal consistency. However, two items were right-skewed, and there were indices of redundancy.

**Conclusion:**

The Swedish CancerSupportSource-Caregiver appears to have the potential to identify unmet needs and symptoms of anxiety and depression among Swedish family members of persons diagnosed with cancer. Since the focus of this study was on translating and adapting the tool, further studies in larger samples are warranted to determine the psychometric properties, factor structure, validity, and reliability.

**Supplementary Information:**

The online version contains supplementary material available at https://doi.org/10.1186/s12913-026-15491-1.

## Introduction

Globally, the incidence and prevalence of cancer are expected to rise [1]. In parallel, research predicts an increase in co-affected family members [2]. Family members of persons diagnosed with cancer have a higher risk for psychological and physical illness compared to the general population [3, 4]. This may result in individual and family psychological and economic suffering, societal costs, and a decrease in caregiving capacity [5, 6]. Therefore, family members report they require support during the cancer trajectory [7]. Recent literature conceptualizes supportive care needs as multidimensional and dynamic, encompassing physical, emotional, informational, practical, social, and existential domains that may vary throughout the cancer trajectory. Therefore, comprehensive assessment tools are needed to identify individual support needs and facilitate tailored supportive care interventions [8–10], yet none have been developed in Swedish.

Since there were no available screening tools in Swedish, we performed a review of existing international tools to identify a suitable screening tool for clinical cancer care and translate it into Swedish. First, we assessed the identified tools and questionnaires based on our experience from conducting empirical and questionnaire research on supportive care needs of family members of persons diagnosed with cancer, e-health, and performing psychometric evaluations. To be considered, the tools needed to be *functional*, of satisfactory *quality*, and *implementable*. Being *functional* means the tool needs to be able to identify individual needs and screen for psychological distress. Having satisfactory *quality* refers to the tool undergoing rigorous development and having satisfactory psychometric properties according to the COSMIN checklist [11]. Being *implementable* means it needs to be practical in length, integrable with existing models of cancer care, and applicable in routine care; namely, it should not be burdening for healthcare professionals (HCPs). This includes considering that HCPs in Swedish patient care are not responsible for the care of family members. The CancerSupportSource-Caregiver (CSS-C) by Zaleta et al. [12], developed in the USA, met the established criteria. Having identified an eligible tool, we presented it to a group of stakeholders including clinical cancer nurses, counselors, family members, and persons diagnosed with cancer. The stakeholders assessed the tool as relevant for family members’ supportive care needs and promising for clinical use and subsequently deemed CSS-C as relevant for adaptation to Swedish and the Swedish healthcare context.

The CSS-C is a validated, multidimensional, electronic screening tool of family members’ needs and psychological distress in clinical cancer care [12]. In CSS-C, screening is followed by automated triaging and tailored referral to support services. Family members rate their level of concern on a five-point Likert scale (1 = *Not at all*, 2 = *Slightly*, 3 = *Moderately*, 4 = *Seriously*, and 5 = *Very seriously*), and for each concern, they also choose support preferences (e.g., “receive additional information,” “speak to an HCP,” or “no action needed”). Administration time ranges from 5 to 8 min.

The CSS-C exists in two versions, one with 33 items [13] and a more recent version with 19 items [12]. In CSS-C, family members self-rate their level of concern about items related to the following domains: patient’s well-being, healthy lifestyle, caregiving tasks, emotional well‐being, and finances. The CSS-C contains an additional item, not included in a domain, that assesses concerns related to the use of tobacco, alcohol, or other substances. The “patient’s wellbeing” domain focuses on the family members’ concerns related to the patient, whereas the remaining domains focus on family members’ concerns related to themselves. The domain “emotional wellbeing” consists of two subscales, each comprising two items intended to indicate symptoms of psychological distress (two items for anxiety and two items for depression). The CSS-C has shown to be feasible, acceptable, and well‐received by family members in an outpatient surgical center [12–14]. Further, the CSS‐C has shown high internal consistency (Cronbach’s alpha = 0.92), a test‐retest reliability of 0.85 [12], and confirmed multi‐dimensionality (inter‐factor correlations ranging from 0.26 to 0.61, representing small to large correlations; although factors are related, each factor is distinct). Moreover, the total distress score of the anxiety and depression subscales strongly correlated with each factor (*rs* = 0.52–0.83; *p* < 0.001). A clinical screening checklist, such as CSS-C, may legitimately include heterogeneous items, but summing all items into a total scale requires stronger evidence.

In our study, we rely on the recommended 19-item version of the tool [12] as we translate, culturally adapt, and pilot evaluate the translated version´s items and domains. Subsequently, an electronic Swedish version of the tool, which includes clinically integrated support services, will be co-designed with stakeholders throughout different design sessions with digital health researchers and family members. Further evaluations in larger samples will follow in future research to determine the psychometric properties, factor structure, validity, and reliability, and will be followed by an evaluation of the feasibility and efficacy of the electronic tool.

## Objective

This study aimed to translate the CancerSupportSource-Caregiver (CSS-C), culturally adapt it, and conduct a descriptive pilot evaluation of the psychometric properties of the Swedish version among Swedish family members of persons diagnosed with cancer. In this study, “family” encompasses the whole family, irrespective of blood or social relations [15]. Given the specific needs of children, this study focuses on family members > 18 years old [16].

## Materials and methods

**Fig. 1:**
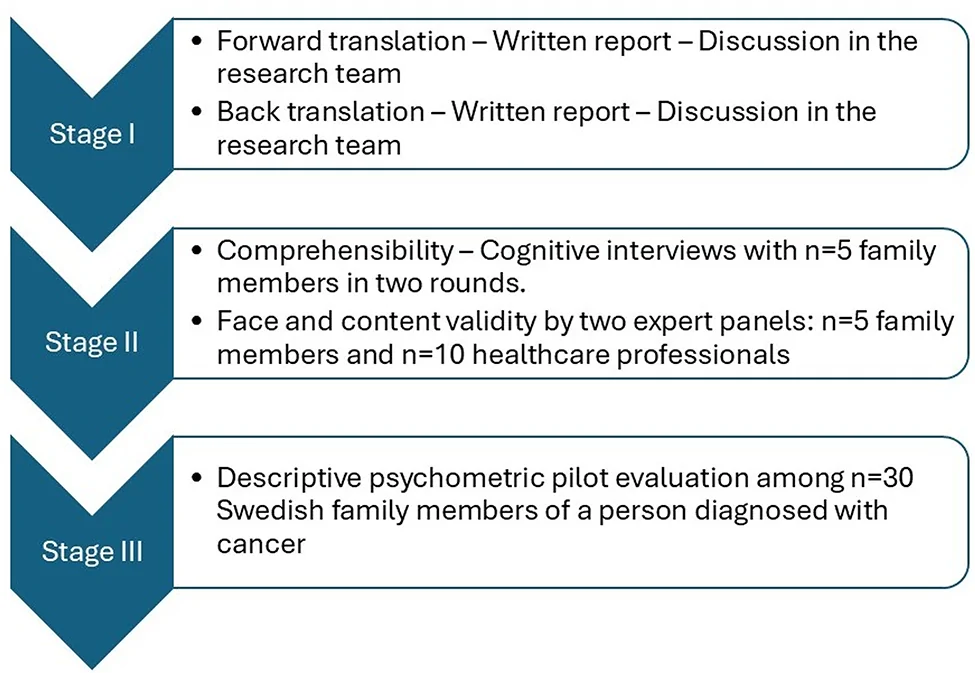
An overview of the translation, cultural adaptation, and pilot evaluation of the preliminary Swedish CSS-C

The translation procedure was informed by, but did not fully adhere to, the recommendations by Beaton and Guillemin [17], as one forward translator and one independent back-translator were used. Permission to translate and culturally adapt the CSS-C was obtained from the original authors [12]. The translation and cultural adaptation involved assessments of comprehensibility and face and content validity by two expert panels comprising family members and HCPs (cancer specialist nurses and clinical social workers), respectively, guided by the recommendations by COSMIN [18, 19] and the ISPOR´s Principles of Good Practice for the Translation and Cultural Adaptation Process for Patient-Reported Outcomes (PRO) Measures [20]. The psychometric properties, validity, and reliability of the translated tool were evaluated using classical test theory, as described by Hobart and Cano [21]. Figure 1 shows an overview of the translation, cultural adaptation, and pilot evaluation processes.

### Data collection

#### Stage I: Translation and cultural adaptation

The CSS-C was (forward) translated into Swedish by a professional translator, who compiled a written report on experienced uncertainties. This step was followed by a discussion among the full research team, who have experience in clinical cancer care and cancer care research, support needs research for family members of persons diagnosed with cancer, questionnaire research, and digital health. Then, another professional translator translated the version (backward) into English, also submitting a written report. Both versions and reports were discussed by the research team until consensus was reached on a first Swedish version, reported in the results section.

#### Stage II: Comprehensibility and face and content validity

The first version was assessed for comprehensibility using cognitive interviews (Supplemental file 1) and the think-aloud method as described by Willis [22]. The interviews were undertaken by the first author, who has experience in cognitive interviewing. Before the first interview, the purpose and procedure of a cognitive interview were repeated to the participants, followed by a short exercise and reflections, as recommended by Willis [21], to facilitate participants’ verbalization of the cognitive process as they described a chosen event. Then, the participants were provided with the translated CSS-C and asked to read each item and its response options and verbalize their thoughts. The first author documented in a protocol the participants’ responses, hesitations, questions, and uncertainties. Interviews were conducted in two rounds with semantic revisions in between, reported in the results section.

The cognitive interviews were followed by a face and content validity assessment (Supplemental file 2 and 3) of the translated CSS-C by an expert panel of HCPs using the Content Validity Index (CVI) according to Polit and Beck [23], reported in the results section. Both family members and HCPs were asked to share their overall impression of the tool and whether they missed any important aspects. After revisions, a new round of CVI was conducted, focusing on the changes made after the first round, with both family members and HCPs. Given the purpose of the study, it was decided not to exclude any items solely based on the results of the CVI.

#### Stage III: Descriptive psychometric pilot evaluation

To pilot test whether the translated and culturally adapted CSS-C appeared to be able to identify family members’ support needs, we collected data from a sample of Swedish family members of persons diagnosed with cancer, who responded to the Swedish CSS-C and sociodemographic questions. The translated and culturally adapted CSS-C´ anxiety and depression subscales were pilot evaluated for convergent validity. Therefore, the participants were also asked to answer the Hospital Anxiety and Depression Scale (HADS) [24].

### Sample size

#### Stage II

The sample size for the expert panels of family members and HCPs was 5–10 persons in accordance with Lynn [25].

#### Stage III

Following recommendations [17, 26], the estimated sample size for the descriptive psychometric pilot evaluation was 30 persons.

### Participants

#### Stage II

The inclusion criteria for the expert panel of family members were being a family member of a person diagnosed with cancer, being over 18 years old, and being able to read and understand Swedish. The inclusion criteria for the expert panel of HCPs were being a healthcare professional with at least one year of professional experience in cancer care.

#### Stage III

The inclusion criteria for participating in the psychometric pilot evaluation of the Swedish CSS-C were being a family member of a person diagnosed with cancer, being over 18 years old, and being able to read and understand Swedish.

### Recruitment

#### Stage II

Family members were recruited using snowball sampling from a patient and next of kin organization via gatekeepers (nurses not involved in the study). We contacted those interested and provided verbal and written information. All participants gave verbal informed consent before the interviews started and before answering the CVI questionnaire. Recruitment proceeded until the estimated sample size was reached and no further uncertainties were discovered during the interviews.

The HCPs were invited using snowball sampling from a national governmental organization for cancer care: Regionala Cancer Centrum. Verbal and written information were provided to prospective participants via a gatekeeper (also a nurse not involved in the study). We contacted those interested and restated the study information. All participants gave written informed consent before answering the CVI questionnaire. Recruitment proceeded until the estimated sample size was reached.

#### Stage III

Family members were recruited via posters in waiting rooms in five oncological/surgical clinics during November 2024 to February 2025. The posters included a QR code leading to a survey tool provided by the university and information about the study aim, procedure, voluntariness, and contact information for the research team, as well as a consent form. Those interested could choose to proceed and, after giving their informed consent, respond to a sociodemographic questionnaire, the Swedish CSS-C, and the HADS.

### Analysis

#### Stage II

Comprehensibility, face validity, and content validity were analyzed per item and for the scale as a whole [11, 23, 27]. With panels of five experts, the threshold for excellent content validity was set at 1.0, and with panels of 10 experts, it was set at 0.78 in accordance with Lynn [25].

#### Stage III

Psychometric analyses of the Swedish CSS-C were performed using IBM SPSS Statistics for Windows, Version 28.0. (Armonk, NY: IBM Corp). Descriptive statistics (i.e., means and standard deviations) were used to evaluate data quality, scaling assumptions, and targeting [28]. The anxiety and depression subscales was evaluated for convergent validity [27] and reliability through internal consistency [27, 28]. A level of statistical significance was set at *p* < 0.05.

### Data quality

Data quality was evaluated based on the proportion of missing data per item and the percentage of computable scale scores, since these determine respondents’ understanding of an item and the extent to which an instrument can be used successfully in a clinical setting [28].

### Scaling assumptions

In Likert scales, items can be summed if they have roughly equivalent means and standard deviations [28]. Further, within a domain, all items should contribute equally and substantially (*r* ≥ 0.30) to the total score. Correspondingly, item response distributions of the domains were reviewed, and item-total correlations were calculated.

### Targeting

To evaluate whether the Swedish CSS-C could target the full variance within the sample, we calculated ceiling and floor effects and skewness. Ceiling and floor effects were considered present if the proportion of response alternatives was > 90% per item and the skewness ranged outside − 1 to 1 [28, 29].

### Internal validity

Based on recommendations [28], internal validity was evaluated by determining whether the domains measured similar but distinct aspects of the construct. Thus, intercorrelations were calculated using Pearson’s correlation coefficient and expecting a moderate correlation (*r =* 0.30–0.70).

### Convergent validity

We evaluated the convergent validity of the subscales intended to indicate symptoms of anxiety and depression. We used Spearman’s rank correlation coefficients to examine associations between the total scores of the Swedish CSS-C anxiety and depression subscales and the corresponding HADS anxiety and depression subscale scores. In accordance with Swinscow [30], a significant (*p* = 0.05) correlation of ≥ 0.40 was expected.

The HADS is a 14-item scale measuring the number and severity of anxiety and depression symptoms in two subscales (seven items per subscale) using a four-point Likert scale [24]. The scale ranges from zero (*not at all*) to three (*very much*), with a total score ranging from 0 to 21. Higher scores indicate more symptoms of anxiety or depression. Cronbach’s alpha values for the HADS ranged between 0.71 and 0.90 for the two subscales. The questionnaire has been validated in several populations [31], including persons diagnosed with cancer [32] and their family members [24].

### Internal consistency

Following Hobart et al. [28] the internal consistency of the Swedish CSS-C and its domains was evaluated using corrected item-total correlations, Cronbach’s alpha, and homogeneity. In accordance with Ware and Gandek [29], the cut-off criteria were > 0.40 for satisfactory item-total correlations, 0.70 for Cronbach’s alpha, and > 0.30 for homogeneity.

### Ethical considerations

This study was conducted following the principles of the Declaration of Helsinki [33] and has been approved by the Swedish Ethical Review Authority (Reg nr 2024-03804-01,2025-01530-02). Participation was voluntary, and informed consent was obtained from all participants. Data were collected and stored in a de-identified form, and no personally identifiable information was included in the dataset used for analysis. Confidentiality was ensured throughout data collection, storage, and analysis. All data were securely stored on password-protected servers at Malmö University, with access restricted to authorized members of the research team.

## Results

### Translation and cultural adaptation

During the translation, we decided to adapt the items “Managing health insurance and medical bills” and “Managing household finances” for cultural reasons. Due to the Swedish tax-financed healthcare system and the fact that family members of cancer patients report such support as not suitable in the Swedish context [7, 34], these items were reframed as *Managing the household* and *Household finances*. Table 1 provides an overview of the items and the domains of the original CSS-C and the revised preliminary Swedish CSS-C.

**Table 1:** Overview of the items and the domains of the original CSS-C and the revised preliminary Swedish CSS-C

Original CSS-C	Swedish CSS-C
**Patient wellbeing**	**Patient wellbeing**
The patient’s eating and nutrition	No changes
Changes in the patient’s mood or behavior	
Changes in the patient’s memory or thinking	
The patient’s pain or physical discomfort	
**Healthy lifestyle (original)**	**Healthy lifestyle**
Eating and nutrition	Eating and nutrition
Exercising and being physically active	Exercising and being physically active
Keeping up with your health care needs	Getting enough sleep^†^
	Your own health^†^
	Keeping up with your health care needs
**Caregiving tasks**	**Caregiving tasks**
Providing transportation to treatment and appointments	*No changes*
Making treatment decisions	
Coordinating medical care for the patient	
Providing physical or medical care to the patient	
**Emotional wellbeing**	**Emotional wellbeing**
Changes or disruptions in work, school, or home life	Changes or disruptions in work, school, or home life
Feeling sad or depressed^d^	Feeling sad or depressed
Feeling nervous or afraid^a^	Feeling nervous or afraid
Worrying about the future and what lies ahead^a^	Worrying about the future and what lies ahead
Feeling lonely or isolated^d^	Feeling lonely or isolated
	Feelings of fatigue^†^
**Finances**	**Family life (new domain)**
Managing household finances	Your relationship^†^
Managing health insurance and medical bills^††^	Managing the household^†^
	Managing household finances
	Other family members’ wellbeing^†^
Tobacco, alcohol, or other substance use	Tobacco, alcohol, or other substance use

^†^Added to the preliminary Swedish version based on feedback from expert panels of healthcare professionals and family members; ^††^Excluded item; ^d^depression subscale; ^a^anxiety subscale

### Comprehensibility

In the first round of cognitive interviews, the five participating family members perceived the Swedish CSS-C as overall comprehensible. Nevertheless, based on the interviews Items 19–23 were reworded to better correspond with the other response options (e.g. *Feeling sad or depressed* was reworded to *Feelings of sadness or depression*). Based on suggestions from the ten HCPs, Item 16, *Household finances*, was changed to the more direct *Finances* (*din/er ekonomi*). In the second round of cognitive interviews conducted with the same five family members, and five of the HCPs, no further uncertainties were identified by the participants why interviewing stopped, as recommended by Willis [21].

### Face and content validity

The expert panels comprised five family members (partners or adult children of persons diagnosed with lung, breast, and colorectal cancer) and ten HCPs (cancer specialist nurses and clinical social workers). Overall, the face validity was deemed satisfactory by both panels.

In the first round of content validity, family members’ I-CVI was 1.0 across items. The S-CVI/UA and the S-CVI/Ave were also 1.0, indicating excellent content validity. The HCPs’ I-CVI ranged from 0.6 to 1.0, and it was below the threshold value for three items: 0.6 for Item 7 (*Exercising and being physically active*) and 0.7 for Item 10 (*Keeping up with your health care needs*) and Item 19 (*Tobacco*,* alcohol*,* or other substance use*). HCPs’ S-CVI/UA was 0.75 and the S-CVI/Ave was 0.88, indicating satisfactory content validity. Since the purpose of implementing the tool in clinical cancer care is to prevent negative health outcomes among family members, and these items were deemed relevant by the expert panel of family members, none were excluded before psychometric evaluation.

The cognitive interviews revealed that the following aspects were missing: (a) the impact of cancer on *family relationships*, (b) concerns about *own health*, (c) concerns about *own sleep*, (d) feelings of *fatigue*, and (e) concerns about *other family members*. These aspects are supported by research on family members’ needs during the cancer trajectory [35]. Therefore, they were added to the Swedish CSS-C, leading to the creation of a new domain (Table 1), which was presented to the two expert panels and deemed comprehensible. The second round of I-CVI assessment of the added items showed an I-CVI of 1.0 for all items in both panels. As a result, the translated and adapted preliminary Swedish CSS-C version comprises 24 items. Table 1 shows an overview of items and domains in the original CSS-C and the revised preliminary Swedish CSS-C. The authors of the original CSS-C were informed of all changes.

### Psychometric pilot evaluation, validity, and reliability

In total, 30 family members were recruited via convenience sampling to respond to the Swedish version of the CSS-C. Participants’ characteristics are shown in Table 2. The evaluations of the psychometric properties of the Swedish CSS-C are presented in Table 3. The pilot evaluations of the domains of the Swedish CSS-C are shown in Table 4. Further, the evaluations of the convergent validity of the anxiety and depression subscales are shown in Table 5.

**Table 2:** Characteristics of the sample of Swedish family members participating in the descriptive psychometric pilot evaluation

		Number	%
**Sex**	Female	25	83.3
	Male	4	13.3
	Other	0	0
	Missing	1	3.3
**Age in years**			
	20–30	1	3.3
	31–40	2	6.7
	41–50	13	43.3
	51–60	10	33.3
	61–70	3	10.0
	71–80	1	3.3
	81 and above	0	0
**Education**			
	Nine-year compulsory school	0	0
	Upper secondary school	8	26.7
	Higher education	22	73.3
**Relation**			
	Partner	19	63.3%
	Child	7	23.3%
	Sibling	2	6.7%
	Parent	2	6.7%

**Table 3:** Pilot evaluations of the psychometric properties of the preliminary Swedish 24-item CSS-C among *n* = 30 Swedish family members of persons diagnosed with cancer

Item	Item frequency distribution in %						Item descriptive statistics		
	Missing data *n* (%)	Not at all	Slightly	Moderate	Seriously	Very Seriously	Mean	SD	Skewness
**First, we want to understand your concerns about THE PATIENT** **Today, how CONCERNED are you about…**									
1. The patient’s eating and nutrition	0	40	16.7	20	10	13.3	2.40	1.453	0.610
2. Changes in the patient’s mood or behavior	0	13.3	30	16.7	26.7	13.3	2.96	1.299	0.065
3. Changes in the patient’s memory or thinking	0	26.7	26.7	20	20	6.7	2.53	1.279	0.344
4. The patient’s pain or physical discomfort	0	6.7	30	26.7	10	26	3.20	1.324	0.180
5. Your relationship^†^	1 (3.3%)	40	10	6.7	30	10	2.59	1.547	0.200
**Next**,** we want to understand your concerns about YOURSELF** **Today**,** how CONCERNED are you about…**									
6. Eating and nutrition	0	56.7	20	10	10	3.3	1.83	1.177	1.297
7. Exercising and being physically active	0	33.3	30	13.3	16.7	6.7	2.33	1.295	0.650
8. Getting enough sleep^†^	0	16.7	20	26.7	16.7	20	3.03	1.377	0.021
9. Your own health^†^	0	23.3	30	23.3	10	13.3	2.60	1.329	0.525
10. Keeping up with your health care needs	0	36.7	26.7	13.3	13.3	10	2.33	1.373	0.717
11. Providing transportation to treatment and appointments	0	40	23.3	23.3	6.7	6.7	2.17	1.234	0.841
12. Making treatment decisions	0	13.3	30	36.7	10	10	2.73	1.143	0.418
13. Coordinating medical care for the patient	0	16.7	36.7	26.7	10	10	2.60	1.192	0.597
14. Providing physical or medical care to the patient	0	33.3	26.7	26.7	6.7	6.7	2.27	1.202	0.723
15. Managing the household^†^	0	40	30	10	10	10	2.20	1.349	0.964
16. Managing household finances	0	26.7	23.3	30	6.7	13.3	2.57	1.331	0.427
17. Other family members’ wellbeing^†^	0	6.7	23.3	26.7	13.3	30	3.37	1.326	− 0.072
18. Changes or disruptions in work, school, or home life	0	6.7	26.7	23.3	26.7	16.7	3.20	1.215	− 0.040
19. Feeling sad or depressed^d^	0	3.3	23.3	20	30	23.3	3.47	1.196	− 0.240
20. Feeling nervous or afraid^a^	0	3.3	6.7	26.7	30	33.3	3.83	1.085	− 0.687
21. Worrying about the future and what lies ahead^a^	0	0	0	23.3	30	46.7	4.23	0.817	− 0.420
22. Feeling lonely or isolated^d^	0	16.7	26.7	10	13.3	33.3	3.20	1.562	− 0.065
23. Feelings of fatigue^†^	1 (3.3%)	13.3	16.7	23.3	23.3	23.3	3.28	1.386	− 0.276
24. Tobacco, alcohol, or other substance use	0	76.7	16.7	0	3.3	3.3	1.40	0.932	2.912

^†^Added to the preliminary Swedish version based on feedback from expert panels of healthcare professionals and family members. ^d^Depression subscale ^a^Anxiety subscale

**Table 4:** Pilot evaluations of the domains of the preliminary Swedish CSS-C among *n* = 30 Swedish family members of persons diagnosed with cancer

Domains	Mean	SD	Item Total Correlations	Scale Mean If Item Deleted	Scale Variance If Item Deleted	Cronbach’s Alpha If Item Deleted
**Patient wellbeing**						
The patient’s eating and nutrition	2.40	1.453	0.726	8.70	8.976	0.652
Changes in the patient’s mood or behavior	2.97	1.299	0.656	8.13	10.257	0.696
Changes in the patient’s memory or thinking	2.53	1.279	0.419	8.57	12.047	0.809
The patient’s pain or physical discomfort	3.20	1.324	0.570	7.90	10.714	0.739
	Cronbach’s alpha 0.78 Internal homogeneity 0.60					
**Healthy lifestyle**						
Eating and nutrition	1.83	1.177	0.779	10.30	21.252	0.880
Exercising and being physically active	2.33	1.295	0.723	9.80	20.855	0.890
Getting enough sleep*	3.03	1.377	0.703	9.10	20.438	0.895
Your own health^†^	2.60	1.329	0.808	9.53	19.775	0.872
Keeping up with your health care needs	2.33	1.373	0.795	9.80	19.545	0.875
	Cronbach’s alpha 0.90 Internal homogeneity 0.72					
**Caregiving tasks**						
Providing transportation to treatment and appointments	2.17	1.234	0.624	7.60	9.834	0.864
Making treatment decisions	2.73	1.143	0.669	7.03	10.033	0.844
Coordinating medical care for the patient	2.60	1.192	0.799	7.17	9.040	0.791
Providing physical or medical care to the patient	2.27	1.202	0.768	7.50	9.155	0.804
	Cronbach’s alpha 0.86 Internal homogeneity 0.71					
**Emotional wellbeing**						
Changes or disruptions in work, school, or home life	3.20	1.215	0.756	18.00	27.655	0.908
Feeling sad or depressed	3.47	1.196	0.884	17.73	26.478	0.890
Feeling nervous or afraid	3.83	1.085	0.826	17.37	28.102	0.900
Worrying about the future and what lies ahead	4.23	0.817	0.707	16.97	31.620	0.919
Feeling lonely or isolated	3.20	1.562	0.789	18.00	24.207	0.909
Feelings of fatigue^†^	3.27	1.363	0.775	17.93	26.133	0.906
	Cronbach’s alpha 0.92 Internal homogeneity 0.73					
**Family life** ^†††^						
Your relationship^†^	2.59	1.547	0.420	8.28	8.564	0.582
Managing the household^†^	2.21	1.373	0.417	8.66	9.377	0.580
Managing household finances	2.62	1.321	0.511	8.24	8.975	0.515
Other family member´s wellbeing^†^	3.45	1.270	0.357	7.41	10.251	0.619
	Cronbach’s alpha 0.64 Internal homogeneity 0.48					

^†^Added to the preliminary Swedish version based on feedback from expert panels of healthcare professionals and family members. ^†††^New domain in the preliminary Swedish version

### Data quality

The proportion of missing data per item was low (3.3%) and distributed across items (Item 5, *Relation*, and Item 23, *Fatigue*). All items were responded to by 97% of the participants. The computable scale scores were 100%. This indicates high data quality.

### Scaling assumptions

The item means of the total scale ranged from 1.40 to 4.23, with responses distributed across all options (Table 4). Means across domains ranged from 1.83 to 4.23 (Table 4). All items contributed substantially to their allocated domain (> 0.30), indicating items can be summed.

### Targeting

According to the predefined criterion for floor and ceiling effects (> 90% of responses in a single response category), no floor or ceiling effects were observed (Table 3). Frequency distributions for most items were distributed across response categories. However, Item 24 (*Tobacco*,* alcohol*,* or other substance use*) showed a pronounced concentration of responses in the lowest response category, with 76.7% of participants selecting “Not at all” and demonstrated substantial positive skewness. Item 6 (Eating and nutrition) also showed positive skewness. Overall, scale skewness was 0.39, indicating acceptable targeting at the scale level.

### Internal validity

The correlations for the internal validity of the original domains ranged from 0.419 to 0.885, and the Swedish CSS-C ranged from 0.357 to 0.884, both indicating redundancy (Table 4).

### Internal consistency

Cronbach’s alpha value was 0.93 for the total Swedish CSS-C scale (Table 4). Across original domains, Cronbach’s alpha values ranged from 0.78 to 0.92, which indicates a need for further testing in a larger representative sample. Likewise, the Cronbach’s alpha value for the new “family life” domain was 0.64, which is below the threshold. Across all domains, the average item correlations ranged from 0.48 to 0.72, indicating a satisfactory internal consistency of the domains. The item-total correlations exceeded 0.30, although 14 items exceeded 0.70, indicating redundancy. In addition, for Item 3 (*Changes in the patient’s memory or thinking*), the alpha value if the item was deleted exceeded the alpha value of the domain, indicating unresolved problems with this item in its allocated domain.

### Convergent validity

As expected, significant positive correlations were found between the total score of the Swedish CSS-C and the corresponding HADS anxiety and depression subscales (*p* < 0.001). The correlation between the Swedish CSS-C Anxiety subscale and the HADS Anxiety subscale was associated with a 95% confidence interval (CI) ranging from 0.46 to 0.86, while the correlation between the Swedish CSS-C Depression subscale and the HADS Depression subscale had a 95% CI ranging from 0.57 to 0.89. These findings support the convergent validity of the Swedish CSS-C. The correlations are presented in Table 5.

**Table 5:** Correlations between preliminary Swedish CSS-C’s and the HADS’s anxiety and depression subscales

	Swedish CSS-C Anxiety subscale	Swedish CSS-C Depression subscale
HADS Anxiety subscale	*r*=0.652 *p* = < 0.001, 95% CI 0.46 0.86	-
HADS Depression subscale	-	*r*=0.779, *p* = < 0.001, 95% CI 0.57 0.89

## Discussion

Following established guidelines [20] and previous research on the translation and adaptation of supportive care needs assessment tools [36, 37], the translation, cultural adaptation, and psychometric evaluation of the Swedish CSS-C followed a multi-step process. In this study, the CSS-C was translated from English into Swedish and culturally adapted for Swedish family members of persons diagnosed with cancer and for the Swedish healthcare context. This work will be followed by further evaluation of the Swedish CSS-C’s psychometric properties, including its factor structure, validity, and reliability, in a larger sample.

In the present study, the expert panel of family members found the translated version to be comprehensible, and the content was deemed relevant by both the expert panel of family members and that of the HCPs. However, the two expert panels requested additional items (Table 1). We considered the balancing act [27] between a questionnaire’s practical length, satisfactory measurement properties, and representation of all relevant aspects of the phenomena under study, while keeping in mind the intended use and purpose of the Swedish CSS-C—namely, trying to prevent negative health outcomes among family members. The suggested items were added to the Swedish version because they cohered with the literature on family members’ support needs [35], showed excellent content validity among the experts of this study, and there were indications of promising psychometric properties (Tables 3 and 4). The items *Relation* and *Fatigue* were included in the 33-item version of the CSS-C but were excluded during factor analyses [12]. *Own health* was added upon request from the expert panels, although from a measurement perspective, including an item that intends to directly measure the latent construct of that domain (i.e., concerns about healthy lifestyle) may be problematic. Noticeably, there were indices of redundancy across domains that need further evaluation in a larger sample.

Similarly, during the cross-cultural adaptation, based on the expert panels’ feedback and empirical research, we created a new domain labeled “family life” (Table 1). The added items within that domain showed excellent content validity among the experts of this study and satisfactory psychometric properties (Tables 3 and 4). However, the domain showed a lower level of internal consistency than the other domains. This finding corresponds with findings related to another screening tool of family members of cancer patients’ supportive care needs—the Swedish Supportive Care Needs Survey – Partners and Caregivers [34]. This tool comprises the domains “health care service needs,” “emotional and psychological needs,” “informational needs,” and “work and social needs,” and its psychometric evaluations show content validity problems with the “work and social needs” domain. Accordingly, the difficulties in grouping the social dimensions of the Swedish family caregiving experience seem relevant for future research to explore. Nevertheless, the “family life” domain showed a Cronbach’s alpha value of 0.64, which is just below the threshold, and the internal homogeneity was encouraging (0.48). As a result, the domain was kept in its current form until further evaluations are conducted in a larger sample.

The items *Tobacco*,* alcohol*,* or other substance use* and *Eating and nutrition* (the family members’ concern for themselves) were right skewed (2.912 and 1.297, Table 4). Although the predefined criterion for a floor effect was not reached, the distribution of responses for the substance-use item, with 76.7% of respondents selecting the lowest response category, suggests a tendency towards a floor effect and should therefore be interpreted with caution. To some degree, the skewness of the item *Tobacco*,* alcohol*,* or other substance use* may be the result of the underreporting of such use identified in previous research [36], and since the purpose of translating and adapting the CSS-C is to prevent negative health outcomes, we decided to keep this item. Regarding the item *Eating and nutrition*, we found no evident empirical explanation for its skewness apart from the presumption that family members might put their own needs aside [7], but at the same time, this would show also in related items. Hence, this item may not be useful for measuring Swedish family members’ needs for support. However, we found that excluding this item requires an evaluation in a larger sample.

Lastly, the “emotional well-being” domain showed satisfactory results for measuring family members’ needs for emotional support (Tables 3 and 4). Satisfactory measurement properties of this domain were considered important since the literature shows a correspondence between unmet emotional needs and negative health outcomes [37], and the clinical use of the full electronic CSS-C with tailored support services has been shown to improve emotional well-being among family members [13]. The observed associations with the corresponding HADS subscales provide preliminary support for the convergent validity of the anxiety- and depression-related items in the Swedish CSS-C. These findings suggest that concerns related to psychological distress may be captured by the instrument. However, these findings do not establish the ability of the subscales to independently screen for or diagnose anxiety and depression. Importantly, this is not the final version of the tool, and the subdomains are not yet fully established, indicating that the findings are preliminary and should be interpreted with caution. Further evaluations in larger samples are warranted to determine the psychometric properties, factor structure, validity, and reliability of the Swedish CSS-C.

### Methodological considerations

A limitation of this study is that the translation process involved one forward translator and one back-translator rather than multiple translators as recommended in established cross-cultural adaptation guidelines. However, cognitive interviews with members of the target population provided an additional opportunity to evaluate the linguistic and conceptual equivalence of the translated instrument. Further, we used classical test theory to pilot evaluate the psychometric properties of the Swedish CSS-C. In a larger sample, the application of, for instance, the Rasch measurement theory would have provided a deeper understanding of the function of items and response options [21]. Nevertheless, classical test theory offers well-established methods and criteria for the evaluation of validity and reliability in the sample sizes recommended for pilot evaluations of translated and culturally adapted questionnaires [17]. We only undertook a preliminary evaluation of the translation and adaptation in a small sample; thus, we did not perform exploratory or confirmatory factor analyses, which restricts the evaluation of the tool’s underlying structure and construct validity. Hence, evaluations in a larger sample are warranted to verify the factor structure of the Swedish CSS-C. Another consideration is that we used an anonymous public survey, which entails the possibility of multiple answers by a single person. On the other hand, it enabled the collection of data without burdening the HCPs at the cancer clinics. Nevertheless, further evaluations in a larger and more representative sample are warranted.

## Conclusion

In this study, we translated and culturally adapted the CSS-C and conducted a descriptive pilot evaluation of the Swedish version to assess the success of the translation. Overall, the translated version is promising but requires further validation in a larger, representative sample. The preliminary Swedish 24-item CSS-C was deemed comprehensible, content relevant, and promising for clinical use by two expert panels of family members and HCPs. Further, evaluations of the Swedish CSS-C´s psychometric properties yielded encouraging results in a sample of Swedish family members of persons diagnosed with cancer. The identified indices of redundancy and skewness among the items need further evaluation. Since the aim of this study was to translate and adapt the CSS-C to Swedish, future studies should conduct further psychometric analysis in larger samples to assess the factors, validity, and reliability of the Swedish CSS-C.

### Clinical implications

The preliminary findings suggest that the Swedish version of CSS-C has the potential to serve as a clinically useful screening checklist for identifying unmet supportive care needs among family members of persons diagnosed with cancer. Early identification of concerns may enable HCP´s to provide timely, tailored interventions and referrals before these needs result in adverse physical or psychological health outcomes. Although the Swedish CSS-C seems to be applicable for clinical use, the current findings are preliminary. Until further psychometric testing is completed in larger and more representative samples, results should be interpreted with caution, and the tool should be used as a complement to, rather than a replacement for, clinical assessment and dialogue with family members.

## Supplementary Information

Below is the link to the electronic supplementary material.


Supplementary Material 1



Supplementary Material 2



Supplementary Material 3


## Data Availability

The datasets used during the current study are available from the corresponding author on reasonable request.

## Abbreviations

CSS-C: Cancer support source – Caregivers
HADS: Hospital Anxiety and Depression Scale
HCP: Healthcare professionals
I-CVI: Item content validity
S-CVI/Ave: Average scale content validity
S-CVI/UA: Universal agreement scale content validity
