# Supplementary material for: Translation, cultural adaptation, and descriptive psychometric pilot evaluation of the Swedish Cancer Support Source-caregiver
Source: BMC Health Serv Res. 2026 Aug 31;26:1216. doi: 10.1186/s12913-026-15491-1 (PMC13531802; doi:10.1186/s12913-026-15491-1)
Supplement: Supplementary file 1 [file 12913_2026_15491_MOESM1_ESM.docx]

**Supplemental file 1. Cognitive interviews**

**Opening questions:**

**Could you please read the first item and the response options out loud and verbalize your thoughts?** The interview then followed the items of the translated CSS-C questionnaire, and each item were assessed as follows:

Comprehension

- Can you tell me in your own words what this question is asking?
- What does the term/phrase "[e.g, involment in care]" mean to you?
- Was anything in this question unclear or difficult to understand?

Retrieval

- What were you thinking about when answering this question?
- What did you think about when answering this question?

Judgment

- How easy or difficult was it to decide on your answer?
- Was there anything you were unsure about when answering this question?

Response

- Were the response options easy to use?
- Was there a response option you would have preferred but was not available?

**Closing Questions**

- Were any questions difficult, confusing, or uncomfortable to answer?
- Were there any words or phrases that should be revised?
- Do you have any other comments or suggestions regarding the questionnaire?

**Follow-up questions**

Probing and prompting questions were used e.g. *Could you explain a bit further? Could you give and example? Why do you think that is?*
