# Supplementary material for: Translation, cultural adaptation, and descriptive psychometric pilot evaluation of the Swedish Cancer Support Source-caregiver
Source: BMC Health Serv Res. 2026 Aug 31;26:1216. doi: 10.1186/s12913-026-15491-1 (PMC13531802; doi:10.1186/s12913-026-15491-1)
Supplement: Supplementary file 2 [file 12913_2026_15491_MOESM2_ESM.docx]

Frågeformulär för att värdera innehållsvaliditet av frågeformuläret

The Cancer Support Source – Caregivers (CSS-C)

Behovsskattningsverktyget CSS-C syftar till att identiﬁera närståendes behov av stöd då en familjemedlem är diagnostiserad med cancer. Vi vill be dig att gradera enkätfrågornas relevans med tanke på syftet.

**Var god och kryssa i det svarsalternativ som du tycker passar in bäst.**

| **Fråga i enkäten** | **Ej relevant** | **Till viss del relevant** | **Ganska relevant** | **Mycket relevant** |
| --- | --- | --- | --- | --- |
| Din närståendes mat- och näringsintag |  |  |  |  |
| Förändringar i din närståendes humör eller beteende |  |  |  |  |
| Förändringar i din närståendes minnes- eller tankeförmåga |  |  |  |  |
| Smärta eller fysiskt obehag för din närstående |  |  |  |  |
| Ditt mat- och näringsintag |  |  |  |  |
| Din motion och fysiska aktivitet |  |  |  |  |
| Att kunna tillgodose dina egna hälso- och sjukvårdsbehov |  |  |  |  |
| Att kunna ordna transport för din närstående till behandlingar och besök |  |  |  |  |

| Din delaktighet i beslut rörande din närståendes vård och behandling |  |  |  |  |
| --- | --- | --- | --- | --- |
| Att behöva samordna vården för din närstående |  |  |  |  |
| Att behöva vårda din cancersjuka närstående |  |  |  |  |
| Att kunna sköta hushållet |  |  |  |  |
| Din/er ekonomi |  |  |  |  |
| Påverkan på arbete, skola eller vardagsliv |  |  |  |  |
| Känslor av nedstämdhet |  |  |  |  |
| Känslor av oro eller rädsla |  |  |  |  |
| Framtiden och vad som väntar |  |  |  |  |
| Känslor av ensamhet och isolering |  |  |  |  |
