# Supplementary material for: Translation, cultural adaptation, and descriptive psychometric pilot evaluation of the Swedish Cancer Support Source-caregiver
Source: BMC Health Serv Res. 2026 Aug 31;26:1216. doi: 10.1186/s12913-026-15491-1 (PMC13531802; doi:10.1186/s12913-026-15491-1)
Supplement: Supplementary file 3 [file 12913_2026_15491_MOESM3_ESM.docx]

Frågeformulär för att värdera innehållsvaliditet av frågeformuläret

The Cancer Support Source – Caregivers (CSS-C)

Behovsskattningsverktyget CSS-C syftar till att identiﬁera närståendes behov av stöd då en familjemedlem är diagnostiserad med cancer. Vi vill be dig att gradera enkätfrågornas relevans med tanke på syftet.

**Var god och kryssa i det svarsalternativ som du tycker passar in bäst.**

| **Fråga i enkäten** | **Ej relevant** | **Till viss del relevant** | **Ganska relevant** | **Mycket relevant** |
| --- | --- | --- | --- | --- |
| Er relation |  |  |  |  |
| Din sömn |  |  |  |  |
| Din egen hälsa |  |  |  |  |
| Andra närståendes mående |  |  |  |  |
| Känslor av orkeslöshet |  |  |  |  |
